# Therapeutic dilemmas with benzodiazepines and Z-drugs: insomnia and anxiety disorders versus increased fall risk: a clinical review

**DOI:** 10.1007/s41999-022-00731-4

**Published:** 2022-12-28

**Authors:** Andreas Capiau, Liesbeth Huys, Eveline van Poelgeest, Nathalie van der Velde, Mirko Petrovic, Annemie Somers, the EuGMS Task, Finish Group on FRIDs

**Affiliations:** 1 Department of Pharmacy, Ghent University Hospital, Ghent, Belgium; 2 Pharmaceutical Care Unit, Faculty of Pharmaceutical Sciences, Ghent University, Ghent, Belgium; 3 Department of Internal Medicine/Geriatrics, Amsterdam Public Health Research Institute, Amsterdam University Medical Centers, Amsterdam, The Netherlands; 4 Department of Internal Medicine and Paediatrics, Faculty of Medicine and Health Sciences, Ghent University, Ghent, Belgium; 5 Department of Geriatrics, Ghent University Hospital, Ghent, Belgium

**Keywords:** Benzodiazepines and Z-drugs, Falls, Older people, Adverse reactions, Potentially inappropriate medication, Deprescribing

## Abstract

**Purpose:**

The aim of this clinical review was to summarise the existing knowledge on fall risk associated with benzodiazepines (BZDs) and Z-drugs in older people with focus on appropriate prescribing, including deprescribing.

**Methods:**

We conducted a literature search in June 2021 in PubMed and Embase with citation and reference checking. Personal reference libraries and international websites were also used. Keywords for the searches included “benzodiazepines”, “Z-drugs”, “falls”, “deprescribing”, “fall-risk-increasing-drugs”, “inappropriate prescribing”, “older people” and matching synonyms. We discuss use of BZDs and Z-drugs, potential fall-related adverse reactions, alternatives for and deprescribing of BZDs and Z-drugs in older persons.

**Results:**

BZDs and Z-drugs differ in fall-related adverse effect profile. They contribute to fall risk through orthostatic hypotension, dizziness and/or imbalance, sedation, muscular weakness, ataxia, etc. Fall incidents contribute significantly to mortality and morbidity. Therefore, there is a need for appropriate prescribing and use of BZDs and Z-drugs in older people. In practice, this means pertaining to a strict indication, strongly consider to non-pharmacological alternatives, limit use to the lowest dose and the shortest duration possible. Judicious deprescribing should be considered and encouraged as well. Practical resources, tools and algorithms are available to guide and assist clinicians in deprescribing BZDs and Z-drugs.

**Conclusions:**

Prescribing BZDs and Z-drugs should be done in a well-considered way in fall-prone older people. A good overview and insight in the fall-related adverse effects of these drugs, as well as the availability of different strategies to increase the appropriate use, including deprescribing initiatives, can assist clinicians in clinical decision-making.

## Key summary points

**Aim:**

To summarise the existing knowledge on fall risk associated with benzodiazepines (BZDs) and Z-drugs in older people with focus on appropriate prescribing, including deprescribing.

**Findings:**

Different mechanisms contribute to increased BZD/Z-drug-related fall risk: orthostatic hypotension, dizziness and/or imbalance, sedation, muscular weakness, ataxia, etc. BZDs and Z-drugs have shown their effectiveness for the short-term symptomatic treatment of insomnia and anxiety in older people. However, prolonged use has been associated with several adverse effects, whereby the risks outweigh the benefits. Different strategies are described to increase the appropriate use of BZDs and Z-drugs including deprescribing initiatives.

**Message:**

BZDs and Z-drugs should be used with caution in older people. A multifaceted approach including non-pharmacological interventions, comprehensive medication reviews, shared decision-making and close interprofessional communication and collaboration is warranted.

## Introduction

Falls are, after road traffic injuries, the second leading cause of injury-related deaths worldwide. Each year, an estimated number of 684,000 individuals die from injurious falls globally [1]. Older age is one of the key risk factors for falls and falls and fall-related injuries are a common problem in older people. In Western Europe, 54,504 older adults died due to falls in 2017. However, death rates varied widely between countries (from 29 to 153 per 100 000) [2]. About one-third of older people (≥ 65 years) fall at least once a year, and this occurs even more frequently in the very old (≥ 80 years), frail and institutionalised patients [3, 4]. Fall incidents in older people are often associated with serious injuries, such as fractures, increased rate of emergency department visits and hospital admissions, leading to increased healthcare expenditures. In addition, falls may lead to loss of independence, loss of self-confidence, increased risk of institutionalisation and decreased quality of life [3, 5].

A multifactorial falls risk assessment is required to identify the individual modifiable risk factors as they may differ between patients [5]. One of the most prominent and also modifiable risk factors is the use of fall-risk-increasing drugs (FRIDs) [6–8]. Fall prevention guidelines recommend performing a medication review to identify the inappropriate use of FRIDs as part of the multifactorial fall prevention strategy [7]. Until recently, there was no consensus about which medications are to be considered as FRIDs. In 2020, a European expert group developed the STOPPFall tool, an explicit screening tool and deprescribing aid in older adults with high fall risk. This tool was created using an expert Delphi consensus process and consists of 14 medication classes [8].

Psychotropic medications, and especially benzodiazepines (BZDs) and Z-drugs (zolpidem, zopiclone, zaleplon), have consistently been reported to increase the risk of falls [6]. A systematic review and meta-analysis by Seppala et al*.* showed an odds ratio (OR) of 1.42 for BZD-related fall risk [6]. Psychotropic drugs are widely used in older people, both in primary and secondary care [9–11]. BZDs and the related Z-drugs are among the world’s most widely prescribed psychotropic drugs in older people. The use of these drugs among community-dwelling older people shows a large variability, ranging from 13.8% to 25.4% in international studies in individual countries, depending on the methodology used. The prevalence is especially high among institutionalised older people [12]. Importantly, their use is associated with multiple potential adverse effects to which older people are more vulnerable due to age-related changes in pharmacodynamics and pharmacokinetics, multimorbidity and drug–drug interactions related to polypharmacy. For example, the altered pharmacodynamics of the ageing brain leads to higher sensitivity to centrally acting agents.

Allosterically binding of BZDs and Z-drugs to the gamma-amino butyric acid (GABA)–benzodiazepine receptor/ion channel complex modulates its activity and thus facilitates GABA-ergic neurotransmission. The GABA–benzodiazepine receptor complex consists of 5 subunits (2 α, 2 β and 1 γ subunits). Each receptor has several isoforms of these subunits. Depending on the affinity of the BZDs for the different isoforms, they have a different pharmacological effect, as schematically illustrated by Griffin et al. and Cheng et al. [13, 14]. For example, a BZD that has a high affinity for the α_2_ subunit will have anxiolytic effects. A BZD with a high affinity for the α_1_ subunit is more likely to cause sedation, amnesia, and antiepileptic effects [13]. There are two central receptors (BZ_1_ and BZ_2_) and one peripheral receptor. BZ_1_ receptors, containing the α_1_ isoform, are predominantly located in the cerebellum, mediating the anxiolytic and hypnosedative actions. BZ_2_ receptors, containing the α_2_ subunit, are located predominantly in the spinal cord and striatum and are involved in mediating the muscle relaxant actions of BZDs. Zolpidem and zaleplon, in contrast to most BZDs and zopiclone, have a high selectivity for the BZ_1_ receptor. They both exhibit sedative effects similar to those of the BZDs, but with a lower probability of undesirable side effects as memory loss and abuse potential [13, 15].

BZDs and Z-drugs are only indicated for the short-term pharmacological management of acute insomnia and for severe and disabling anxiety, the latter as an alternative for selective serotonin reuptake inhibitors (SSRIs) [9, 15]. In older people, about 20–58% suffer from insomnia and the prevalence increases with age [16]. This partially explains the wide use of these drugs in this population. However, the benefits are only marginal and adverse reactions are common. Importantly, the effectiveness on sleep quality diminishes after 4 weeks, while adverse effects persist [17]. A meta-analysis performed by Glass and colleagues revealed that the number needed to harm (i.e., for any adverse reaction) was only six. They concluded that the risks of short-term treatment with BZDs and Z-drugs in older people with insomnia outweigh the potential benefits, especially in patients with high risk for falls [18].

Given the potentially harmful consequences of BZD and Z-drug use, such as falls, there is need for appropriate prescribing and use in older people: pertaining to a strict indication, attention to non-pharmacological alternatives, limitation to the lowest dose and the shortest duration of use possible. Judicious deprescribing should be considered and encouraged as well [19]. It has been reported that deprescribing can be performed safely in older people. To support healthcare professionals in their decision making, the European Geriatric Medicine Society (EuGMS) Task and Finish Group on FRIDs developed a practical deprescribing tool, including FRID drug class specific deprescribing recommendations [7].

In this clinical review, an overview of the current literature regarding use of BZDs and Z-drugs, their fall-related adverse reactions and deprescribing recommendations is provided. This clinical review was informed by a literature search conducted in June 2021 in PubMed and Embase with citation and reference checking. Personal reference libraries and international websites were also used. Keywords for the searches included “benzodiazepines”, “Z-drugs”, “falls”, “deprescribing”, “FRIDs”, “inappropriate prescribing”, “older people” and matching synonyms.

## Fall-related adverse effects of benzodiazepines and Z-drugs

**Fig. 1:**
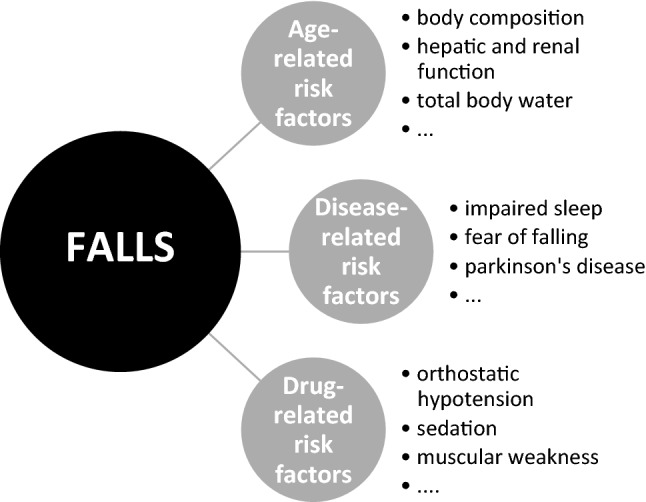
Factors associated with fall risk

Different risk factors contribute to a higher fall-risk, as illustrated in Fig. 1. These factors can be categorised in age-, disease- and drug-related risk factors. Some risk factors are unmodifiable (demographics, diseases), others are potentially modifiable (health and functional conditions, including FRIDs) and should, therefore, be targeted. BZDs and Z-drugs (drug-related risk factor) are associated with falls [4, 6–8, 20, 21], but the underlying reasons for prescribing them (insomnia and anxiety) are also associated with increased fall risk [22]. Therefore, we should be aware for possible confounding by indication in studies addressing the association between use of FRID and fall risk. Three meta-analyses exploring the association between drug use and falls in older people have reported concordant results, with pooled OR for the association between BZDs use and falls ranging from 1.39 (95% confidence interval (CI) 1.24–1.54) to 1.57 (95% CI 1.43–1.72) [23]. BZDs are also strongly associated with hip fractures [18, 24], where approximately one-third of patients with a hip fracture die within a year. Even when falls do not result in fractures, they are associated with fear of subsequent falls, limitation of activities of daily living and consequently decreased quality of life [24]. Z-drugs are often perceived as safer than BZDs. However, use of Z-drugs is also associated with a statistically significant increased risk for fractures (OR = 1.63, 95% CI 1.42–1.87) [25].

Different characteristics of BZDs and Z-drugs may contribute to the risk of falls, such as the elimination half-life, dose and exposure duration. BZDs and Z-drugs are often classified into groups according to elimination half-life (Table 1): long-acting agents (half-life > 20 h), such as diazepam, flurazepam, and nitrazepam; intermediate acting agents (half-life between 10 and 20 h), such as lorazepam, bromazepam, and alprazolam; and short-acting agents (half-life < 10 h), such as lormetazepam, oxazepam and zolpidem [26]. Short-acting BZDs were positively associated with falls during hospital stays in contrast to long-acting BZDs [27, 28]. This is in line with other studies in which the use of short-acting BZDs or Z-drugs was significantly associated with frequent falls [20, 29–31]. In contrast, a prospective cohort study demonstrated that patients using long-acting diazepam were over three times more likely to have a fall in the previous 90 days compared with non-users and all other BZD users. Zolpidem significantly increased the risk of inpatient falls [32]. A possible mechanism to account for the differences in falls risk between different BZDs may include age-related changes in pharmacokinetics and pharmacodynamics in older people which may increase the risk for adverse outcomes caused by BZDs. The changes in pharmacokinetics include changes in body composition, serum albumin, total body water, and hepatic and renal functions [33]. Body fat increases and this can affect the distribution of lipophilic drugs, such as diazepam. Changes in activity of cytochrome P450 (CYP) enzymes, as well as a decrease in albumin plasma levels influence the free fractions of BZDs [34]. Studies reported also an effect on the dose strength on fall risk. BZDs doses > 1 mg/day in diazepam equivalents was significantly associated with falls among hospitalised older people. Accidental falls leading to hospitalisation for femur fractures were more common in patients taking higher doses of BZDs [31, 35]. The study by Ballokova et al*.,* however, did not identify any significant relationship between a history of falls and different doses of BZDs [34]. But, we have to acknowledge the limitation that these self-reported data on past falls may have been biased due to retrospective recall, which would more likely lead to under-estimation rather than over-estimation. Conversely, in a population-based study, Yu et al*.* observed that all dose levels of BZDs and high dose levels of Z-drugs significantly increased the risk of fall-related injuries requiring hospitalisation [20]. This was also confirmed in a study among nursing home residents with dementia. Higher fall risk was already present at low doses and increased further with increasing doses [36]**.** There might be a dose-dependent relationship, but this needs further investigation. Studies have also evaluated the association between duration of BZDs use and falls. Some studies demonstrated that the risk of falls is higher in the first weeks after BZD treatment initiation [21, 37–40] as well as with long-term use (> 28 days) [40]. The study of Carrier et al*.* showed that occasional BZDs use was not associated with increased risk of fall-related fractures. Chronic BZD users had a higher risk of fractures than non-users [21]. However, polypharmacy and the use of multiple types of BZDs and/or Z-drugs or other psychotropics significantly increased the risk of falls [20]. Altogether, it is important to remind the patient that the treatment duration must be limited in time (e.g., < 4 weeks). Therefore, is it useful to indicate a tentative stop date already at initiation of the treatment.

**Table 1:** Elimination half-life of BZDs and Z-drugs

	Half-life (hours)
Long acting agents (half-life > 20 h)	
Clobazam	36
Clorazepaat	50
Diazepam	48
Flunitrazepam	16–35
Flurazepam	24–60
Nitrazepam	30
Nordazepam	55
Prazepam	65
Intermediate acting agents (half-life between 10 and 20 h)	
Alprazolam	12–15
Bromazepam	20
Lorazepam	12–16
Short-acting agents (half-life < 10 h)	
Brotizolam	3–6
Clotiazepam	3–4
Lormetazepam	10
Oxazepam	6–8
Zaleplon	1
Zolpidem	2,4
Zopiclone	5

Source: benzodiazepines, sedatives and anxiolytics SmPC

The therapeutic window for sedative and anxiolytic effects of BZDs and Z-drugs is narrow, and these drugs increase the risk of falls by inducing different mechanisms: sedation and delirium, (orthostatic) hypotension (OH) and dizziness, movement disorders (muscular weakness, extrapyramidal symptoms) and visual disorders [21, 24, 41, 42]. The adverse reaction profile and tolerability is different between BZDs and Z-drugs, but there are also differences within these two classes (Table 2). In clinical practice, selecting a BZD or Z-drug in older people should be individualised, taking into account the adverse reaction profile and tolerability of the drug and patient characteristics. In the following paragraphs, a summary of the literature is provided on effects that might contribute to fall risk caused by BZDs and Z-drugs.

**Table 2:** Prevalence of fall-related adverse reactions of BZDs and Z-drugs

	(Orthostatic) hypotension	Imbalance and/or dizziness	Sedation	Muscular weakness	Visual impairment	Delirium or confusional state	Extrapyramidal symptoms	Ataxia
Benzodiazepines								
Alprazolam	No data	+ + + +	+ + + +	+ +	+ + +	+ + +	+ +	+ + +
Bromazepam	No data	Unknown	Unknown	Unknown	Unknown	Unknown	No data	Unknown
Brotizolam	No data	+ +	+ + +	+	+	+	No data	+
Clobazam	No data	+ + +	+ + +	No data	+ +	+ +	+ + +	+ + +
Clorazepaat	No data	+ + +	+ + + +	+ +	No data	+ +	No data	No data
Clotiazepam	+	+ +	+ +	+ +	+	+ +	+	+
Diazepam	+	+ +	+ + + +	+ +	No data	+ + +	+	+ + +
Flunitrazepam	Unknown	Unknown	Unknown	Unknown	Unknown	Unknown	No data	Unknown
Flurazepam	+	+ + +	+ + +	+ + +	+	Unknown	Unknown	+ + +
Lorazepam	No data	+ + + +	+ + + +	+ + +	No data	+ + +	No data	+ + +
Lormetazepam	No data	+ + +	+ + +	No data	+ + +	No data	No data	No data
Nitrazepam	+	+ + +	+ + +	+ + +	+ + +	+ +	+ +	+ +
Nordazepam	Unknown	+ +	+ + + +	+ +	+ + +	+ +	No data	+
Oxazepam	No data	+ + +	+ + +	+ + +	+ + +	+ + +	No data	+ + +
Prazepam	No data	+ + +	+ + + +	Unknown	+ + +	+ + +	+ + +	+ + +
Triazolam	Unknown	+ + +	+ + +	+	+ +	+ +	No data	+ + +
Z-drugs								
Zaleplon	No data	+ +	+ + +	No data	+ +	+ +	No data	+ +
Zolpidem	+	+ + +	+ + +	No data	+ +	No data	+	+
Zopiclone	No data	+ + +	+ + +	+	+	+	No data	+

+ : < 1/1000 (rare or very rare)

+  + : 1/100 – 1/1000 (uncommon)

+  +  + : 1/10 – 1/100 (common)

+  +  +  + : > 1/10 (very common)

No data: no data known about the adverse effect

Unknown: cannot be estimated from the available data

Source: benzodiazepines, sedatives and anxiolytics SmPC

### Sedation (drowsiness, sleepiness or somnolence)

Sedation is defined as subjective feelings of drowsiness and sleepiness, but also as decreased psychomotor functioning, which can be measured in objective tests [43]. In a meta-analysis of 8 studies, BZDs were more likely than placebo to be associated with complaints of daytime drowsiness [9]. Long-acting BZDs are associated with significant daytime hangover effect, confusion, dizziness, impaired motor coordination, increased risk of falls resulting in fractures, and increased risk of motor vehicle crashes [44]. Sleep inertia, also referred to as sleep drunkenness, is characterised by impaired performance upon awakening from sleep. Such impairments may have implications for fall risk [22]. This can be explained by the decreased metabolism of long-acting BZDs in older people [24] but also by the age-related changes in pharmacodynamics (decreased number of synapses in the brain and their binding function and downward receptor signalling, etc. [45].

### Delirium

BZDs are known to worsen delirium states, especially in older people. The reported prevalence of delirium among older hospitalised patients ranges from 14% to 56%, and almost one-third appeared to be drug-induced [41]. On the other hand, we have to keep in mind, once again, that confounding by indication could be present as BZDs also have their place in treatment guidelines of delirium and acute agitation. The updated Beers [46] and STOPP/START [47] criteria for potentially inappropriate medication use in older people, strongly advise against prolonged BZDs in older people due to increased risk of confusion, delirium, falls, fractures, and motor vehicle crashes.

### Effects on balance

BZDs and Z-drugs exhibit a dose and time-dependent deleterious effect on stability and balance [48]. As balance forms a major fall-risk factor, this supports the hypothesis that balance is the major pathophysiological pathway for BZDs-related fall risk [49]. The effect of BZDs and Z-drugs on balance is most prominent within the first few hours after intake. The impact on balance is more pronounced with higher doses, a shorter time between intake and waking up, or after concomitant use with alcohol or other sedative drugs [50]. Holbrook et al. found a significant increase in adverse events with BZD use. The increase in psychomotor-type adverse reactions found with sedative use in the study of Glass et al. (OR 2.61) is similar to the increase in reports of dizziness and light-headedness found in the Holbrook meta-analysis after BZD use (OR 2.6, 0.7 to 10.3) [9, 18]. Zolpidem binds preferentially with the γ5 subunit of the BZ_1_ receptor as an agonist and does not interfere with muscle coordination (BZ_2_ effect). Zolpidem is also rapid metabolised via CYP3A4 and CYP2C9, resulting in negligible (if any) residual effects even 9 h after intake. Since zolpidem is renally cleared and renal function is reduced in many older people, peak concentrations can be increased [48]. In the same way as for BZD, a trend toward greater fall risk in the early treatment period and with increasing doses has been shown for Z-drugs [49].

### Effects on orthostatic hypotension and postural instability

OH is defined as a reduction in SBP of at least 20 mmHg or DBP of at least 10 mmHg within 3 min of standing up from the supine position, or a similar fall in blood pressure within 3 min of upright tilt table testing to at least 60 degrees [51]. There is an increase in OH prevalence from 15% to 26% with advancing age resulting in more falls, partly due to a decline in autonomic functioning. Aging is also associated with generalised neuronal loss, fewer beta receptors with decreased functionality and a weaker response to catecholamines—all of which are factors contributing to the development of OH [52]. Orthostatic hypotension may play a role in the association between BZDs and falls, since cerebral hypoperfusion may occur, leading to unbalance, gait instability or even transient loss of consciousness. The study by Rivasi et al*.* suggests that older people using BZDs have a higher risk of OH due to an exaggerated immediate (within the first 10 s post-stand) blood pressure drop. The association between BDZs and the immediate blood pressure drop derives from increased venous pooling due to muscular relaxation. Another possible hypothesis includes possible sympathetic hypo-responsiveness resulting from regular use of BZDs [53]. Long-term use of BZDs and Z-drugs was associated with lower DBP and SBP in older, but not in younger, individuals [49]. Postural instability is more pronounced in older patients after BZD administration than in the young. The effect on postural instability is dose-dependent and apparent for BZDs and Z-drugs, but not to the same degree, and depending on the differences in pharmacokinetics (half-life) and affinity for the subtypes of the BZD receptor, GABA_A_ [48].

### Effect on cognition

The acute cognitive effects of BZDs are well known. They can cause short-term cognitive deficits, especially in memory, learning and attention [54]. In addition, some observational studies demonstrated an association between BZDs use and dementia [55, 56]. The association between BZD use and cognitive outcomes can be partially explained by the binding of those molecules to the α_5_ subunit of the BZD receptor in the hippocampus [56]. However, the impact of long-term use of BZDs on cognitive impairment and/or cognitive decline remains unclear and the existing evidence is ambiguous [57]: some evidence indicates an accelerated rate of cognitive decline [58], while other evidence indicates that cognitive decline is transient [59]. The recent review of Ferreira et al*.* suggests an association between BZDs and the development of dementia, especially for agents with long half-life, extended use (the use of a BZD and Z-drug for 6 months or longer during a time period of 1 year regardless of whether the use was daily or infrequent) and earlier exposure (previous initiation of a BZD or Z-drug) [56, 57, 60]. It also seems that, after withdrawal of BZDs, patients recover in many domains of cognitive function, but still remain impaired compared with non-BZD users [61]. Furthermore, even after BZDs are discontinued, the cognitive function of long-term users is reported to remain impaired in most cognitive domains, suggesting possibly irreversible cognitive deficits associated with BZDs use [24]. However, there is need for future studies with proper design to answer this research question.

## Alternatives for benzodiazepines and Z-drugs

BZDs and the related Z-drugs have shown their effectiveness for the symptomatic short-term treatment of insomnia and anxiety in older people [9, 18]. However, as discussed above, the prolonged use has been associated with several adverse effects, whereby the risks outweigh the benefits [18]. In contrast, the benefits of BZDs fade after 4 weeks of use. As a consequence, these medication classes have been included in different explicit criteria for the identification of potentially inappropriate medications (PIMs) in older people [47, 62, 63]. These expert consensus criteria strongly advise against the use of BZDs in older people, especially when used longer than 4 weeks [47, 62, 63]. Explicit criteria and tools are commonly used within the scope of a medication review to identify PIMs use and to prevent, detect and help solving drug-related problems.

An important first step in the management of sleep complaints in older adults is to perform a comprehensive medication review as different drug classes can contribute to insomnia. Examples of insomnia contributing medications are cardiovascular medications (alpha- and beta-blockers, amiodarone, calcium channel blockers, etc.), antidepressants (SSRIs, serotonin–norepinephrine reuptake inhibitors (SNRIs), monoamine oxidase inhibitors), acetylcholinesterase inhibitors, corticosteroids and antimicrobial agents [64, 65]. If treatment is required, a non-pharmacological approach should always be considered first, including relaxation techniques, improving sleep hygiene, and cognitive behavioural therapy for insomnia (CBT-I), such as psychoeducation. Sleep hygiene includes, for example, avoiding daytime naps and limiting substances (such as caffeinated drinks) that adversely affect sleep. CBT-I is the gold standard treatment for chronic insomnia and has demonstrated efficacy in older adults [66–68]. CBT-I focusses on cognitive beliefs and counterproductive behaviours that interfere with sleep. A clinical review by Riemann and colleagues compared the effectiveness of pharmacological treatment with BZDs and psychological and/or behavioural interventions. They conclude that both BZDs hypnotics and psychological interventions are effective to treat insomnia in the short-term (for primary insomnia) but that psychological interventions may be superior for sleep initiation. Moreover, these psychological/behavioural interventions appear to have more durable effects with persistent gains at follow-up, up to 8 months [65, 69]. Another non-pharmacological approach is sleep restriction (restricting the time in bed to the actual sleeping time) [70].

Frequently, melatonin is prescribed as an alternative for BZDs for the treatment of insomnia [71]. Melatonin is a physiological indole amine that modulates circadian rhythms. Melatonin slightly improves sleep onset and sleep duration although the evidence remains inconclusive [72–74]. However, there are concerns about product quality and efficacy which may vary depending on the preparation [64].

Also, for the management of anxiety disorders, a non-pharmacological approach should be considered first. It has been demonstrated that CBT-I substantially reduces worrying and depressive symptoms and improves the general mental health in older patients [75, 76]. If pharmacological treatment is indicated, not BZDs but SSRIs and SNRIs are first choice. These medications have showed positive effects relative to placebo for the treatment of anxiety in older people [77].

Furthermore, if BZD treatment is indicated, it is important to sufficiently inform patients and their carers about the benefit–risk ratio and to discuss both the potential benefits and potential harms.

## Deprescribing of benzodiazepines and Z-drugs

When BZDs and/or Z-drugs are not (longer) indicated, they should be discontinued. However, to avoid the withdrawal syndrome with worsening of insomnia or anxiety symptoms, a gradual reduction is required, as described below. Other common withdrawal symptoms are irritability, restlessness, sweating, headache, muscle cramps, gastrointestinal symptoms, delirium or convulsions [15, 17]. Most often, withdrawal symptoms occur after 12 weeks of habitual use, but tapering is already advised after 4 weeks [17]. Tapering will reduce, but might not eliminate, withdrawal symptoms. Deprescribing is defined as *“the withdrawal of an inappropriate medication, supervised by a healthcare professional with the goal of managing polypharmacy and improving outcomes”* [78] or *“the planned and supervised process of dose reduction or stopping of medication that might be causing harm or no longer providing benefit”* [17]. The aim of deprescribing is to reduce medication burden and harm while maintaining or improving patients’ quality of life. Numerous interventions have been proposed to support deprescribing of BZDs and Z-drugs. However, those strategies are often diverse and/or reported poorly detailed [79]. Deprescribing is challenging, because patients and physicians are often reluctant out of fear for the potential risk of the withdrawal syndrome [80]. In addition, another important barrier for deprescribing, from the patient perspective, is the belief that an alternative effective treatment of their insomnia is lacking [81]. Patients’ personality profile, dose and half-live of BZDs and Z-drugs, duration of treatment and mode of withdrawal have been identified to affect withdrawal success rate [15]. On the other hand, important facilitators to discontinue BZDs are expected improvements in cognition and reductions in other adverse reactions [17].

A systematic review by Rasmussen and colleagues evaluated the barriers and facilitators of different stakeholders when deprescribing BZDs in older people [82]. Patients and physicians were most often targeted in interventions toward deprescribing of BZDs in older people, while relatives, pharmacists or other professional caregivers were often not involved [80, 82]. Remarkably, it was found that patients are willing to deprescribe their BZDs treatment, while doctors consider that patients will resist this [82]. Lack of knowledge, both physical and psychological dependence and patients’ confidence in their physician’s approval to continue BZDs treatment were the most important identified barriers in patients using BZDs. For Z-drugs, identified barriers were factors related to their insomnia (including need for effective treatment of their insomnia), healthcare system factors (including a desire for personalised care), and own positive personal experiences with Z-drugs [81]. Furthermore, nurses and caregivers mentioned the feeling that their options were not valued by physicians and are currently an unused source of support in the deprescribing process [82]. Challenges to deprescribe Z-drugs, from the clinician perspective, were a lack of institutional structures and resources to support deprescribing, the attitudes and practices of previous clinicians, and patient-related factors such as dependence and a lack of alternatives to treat insomnia [81].

In contrast to the general view, studies have shown that deprescribing of BZDs appears to be feasible and safe in older people [83–86]. Importantly, studies show comparable or better quality of life and sleep patterns after deprescribing BZDs [83]. In 2014, Tannenbaum et al*.* showed in the EMPOWER-trial that a deprescribing patient empowerment intervention describing the risk of BZDs use and using a stepwise tapering protocol, resulted in more dose reductions and higher discontinuation rates (27% of the intervention group had discontinued BZD use compared with 5% in the control group) [87]. Patients who did not taper BZD use, mentioned physician’s or pharmacist’s discouragement as the major impediment [87]. Therefore, the D-PRESCRIBE trial was conducted, where the intervention consisted of sending patients an educational deprescribing brochure in parallel to sending their physicians an evidence-based pharmaceutical opinion to recommend deprescribing. This approach yielded in greater discontinuation of prescriptions for PIMs after 6 months [88].

**Fig. 2:**
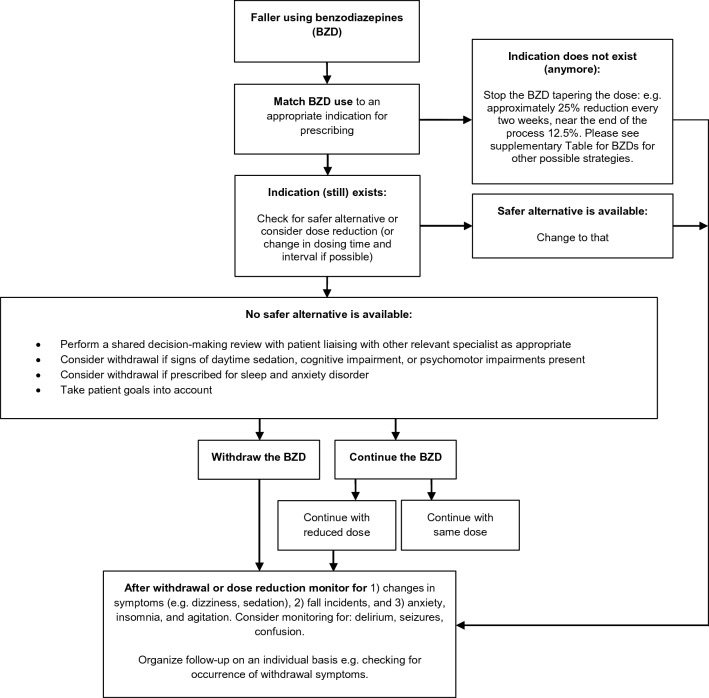
Decision tree for benzodiazepine (BZD) withdrawal in patients who have fallen [8]

It remains unclear whether BZD and Z-drugs deprescribing, and FRID deprescribing in general, is effective as a stand-alone intervention in fall prevention [89]. Nevertheless, fall prevention guidelines emphasise the importance of FRID deprescribing as part of a multifactorial fall prevention strategy. And recently, medication review was confirmed to be an effective component of multifactorial fall preventive interventions in a network meta-analysis [7, 90]. The EuGMS Task and Finish Group on FRIDs developed deprescribing recommendations to facilitate optimal deprescribing in fall-prone older patients, including practical deprescribing decision-trees for 14 drug classes, including BZDs [8]. There is a digital and interactive version of the STOPPFall tool available via https://kik.amc.nl/falls/decision-tree/. Figure 2 displays the STOPPFall decision tree for BZD withdrawal in patients who have fallen. Specific for the withdrawal of BZDs, first of all, the indication of BZDs should be re-evaluated. If there is still an indication present, a safer alternative for BZDs or a dose reduction of BZDs should be considered. If the indication does not (longer) exist, BZD should be stopped in a stepwise manner, by tapering the dose gradually on. For example, a 25% reduction every 2 weeks with a 12.5% reduction near the end of the process. Alternatively, the BZD can be switched to a long-acting equivalent BZD (e.g., diazepam). Each schedule is best tailored to the patient's needs and preferences. To calculate the corresponding mg equivalent dosage of diazepam, an online query ‘CalcBenzo’ can be used http://wiki.psychiatrienet.nl/wiki/Special:RunQuery/CalcBenzo. However, this approach has not been shown to reduce the incidence of withdrawal symptoms or improve cessation rates more than tapering shorter acting BZDs does [17]. As discussed before, a tapering approach will reduce, but might not eliminate withdrawal symptoms. Therefore, during tapering or after withdrawal it is important to monitor patients for changes in symptoms (e.g., sedation, dizziness), fall incidents and anxiety, insomnia and agitation [8]. This monitoring should be planned on a regular basis, for example by a scheduled appointment or through a telephone call [17]. If withdrawal symptoms do occur and at a severity and frequency that is bothersome for the patient, a next dose reduction should be delayed with 1–2 weeks, where after the stepwise tapering can be continued, however, at a slower rate [17].

## Conclusions

Falls and fall-related injuries represent a common problem among older people. Psychotropic medications, and especially BZDs and Z-drugs, have consistently been reported to increase the risk of falls. This clinical review provides an overview of the current literature regarding BZD-related falls and the different fall-related adverse effects of BZDs in older people. The risk of falling is amplified by age, certain diseases and by use of BZDs and Z-drugs which explains why these drugs are considered as potentially inappropriate medications. In this paper, we provide clinicians with alternative treatment strategies, as well as different strategies to increase the appropriate use of BZDs including deprescribing initiatives.

## Data Availability

Data sharing not applicable to this review article as no datasets were generated or analysed during the current study.
